# Loss of Peripheral Sensory Function Explains Much of the Increase in Postural Sway in Healthy Older Adults

**DOI:** 10.3389/fnagi.2017.00202

**Published:** 2017-06-20

**Authors:** Eric Anson, Robin T. Bigelow, Bonnielin Swenor, Nandini Deshpande, Stephanie Studenski, John J. Jeka, Yuri Agrawal

**Affiliations:** ^1^Department of Otolaryngology—Head and Neck Surgery, Johns Hopkins University School of MedicineBaltimore, MD, United States; ^2^Lions Vision Center, Wilmer Eye Institute, Johns Hopkins University School of MedicineBaltimore, MD, United States; ^3^School of Rehabilitation Therapy, Queens UniversityKingston, ON, Canada; ^4^Longitudinal Studies Section, National Institute on AgingBaltimore, MD, United States; ^5^Department of Kinesiology, Temple UniversityPhiladelphia, PA, United States

**Keywords:** vestibular, proprioception, vision, postural sway, aging

## Abstract

Postural sway increases with age and peripheral sensory disease. Whether, peripheral sensory function is related to postural sway independent of age in healthy adults is unclear. Here, we investigated the relationship between tests of visual function (VISFIELD), vestibular function (CANAL or OTOLITH), proprioceptive function (PROP), and age, with center of mass sway area (COM) measured with eyes open then closed on firm and then a foam surface. A cross-sectional sample of 366 community dwelling healthy adults from the Baltimore Longitudinal Study of Aging was tested. Multiple linear regressions examined the association between COM and VISFIELD, PROP, CANAL, and OTOLITH separately and in multi-sensory models controlling for age and gender. PROP dominated sensory prediction of sway across most balance conditions (β's = 0.09–0.19, *p*'s < 0.001), except on foam eyes closed where CANAL function loss was the only significant sensory predictor of sway (β = 2.12, *p* < 0.016). Age was not a consistent predictor of sway. This suggests loss of peripheral sensory function explains much of the age-associated increase in sway.

## Introduction

The ability to stand upright without falling is paramount to the ability to perform many daily tasks. This ability, while appearing to be simple, is in fact quite complex due to the inherent instability of the top heavy human body (Winter, 1995). Visual, somatosensory and vestibular sensory inputs are flexibly fused into a consolidated representation of body position and sway (Hwang et al., 2014). Integration of this sensory information and the appropriate generation of motor commands are necessary for control of upright standing posture in diverse settings and environments (Horak and MacPherson, 1996; Peterka, 2002).

The modified Romberg test (stand on floor and foam with eyes open and then closed) has been described as a test of sensory integration for balance (Cohen et al., 1993; Agrawal et al., 2011). The sensory information that is available and reliable is progressively reduced across the four conditions. On the floor with eyes open (Condition 1), vision, proprioception, and vestibular inputs all provide reliable and consistent information regarding body sway. Closing the eyes (Condition 2) alters the availability of vision. Standing on foam (Condition 3) makes ankle proprioceptive information inconsistent with head motion, resulting in incongruent information about the amplitude and velocity of body sway coming from the ankle proprioceptors relative to the visual and vestibular systems (Patel et al., 2008). Standing on foam with eyes closed (Condition 4) obscures both visual and proprioceptive inputs. As such individuals must rely more on vestibular information to maintain balance (Agrawal et al., 2009; Davalos-Bichara and Agrawal, 2014; Koo et al., 2015).

Older adults have reduced visual, vestibular, and proprioceptive function (Freeman et al., 2008; Li et al., 2015a,b; Deshpande et al., 2016). Age-related visual, proprioceptive, or vestibular loss could make sensory integration for balance more difficult via reduced or less accurate sensory signals (Horak et al., 1990; Horak and Hlavacka, 2001; Black et al., 2008; Ko et al., 2015; Deshpande et al., 2016). Older adults are also known to have increased postural sway and loss of balance, particularly under conditions of reduced availability or reliability of sensory input (Peterka and Black, 1990). Loss of the lower visual field, vestibular disorders, and diabetic peripheral neuropathy have all been independently associated with an increase in postural sway (Black et al., 2008; Freeman et al., 2008; Serrador et al., 2009; Najafi et al., 2010). However, it is unclear how age-related loss of visual, vestibular, and proprioceptive function independently influences postural sway area across a range of balance conditions where sensory information is progressively reduced. Further, it is not known whether the relationships between sensory function and sway area are independent of age.

Here we used data from the Baltimore Longitudinal Study of Aging (BLSA) to investigate the independent association between isolated tests of vestibular, visual, ankle proprioceptive function, and age with postural sway while standing on firm and foam surfaces with eyes open and closed. We hypothesized that there would be a shift to otolith function as the surface became unstable, particularly with eyes closed.

## Methods

The BLSA is an ongoing prospective cohort study initiated by the National Institute on Aging in 1958. Subjects are community-dwelling participants age 20–103 who undergo a standardized array of tests over 3 days every 1–4 years at the National Institute on Aging. This study includes a cross-sectional sample of all BLSA participants seen between August 2014 and December 2015. During this time period 366 participants underwent vestibular and postural sway testing, and of those 321 participants also underwent visual field testing, and 244 participants also underwent proprioception testing. All participants provided written informed consent. The BLSA study protocol was approved by the Institutional Review Board associated with the BLSA at Harbor Hospital. Demographic information was collected along with height, weight, history of smoking, diabetes, and hypertension. A positive smoking history was defined as smoking at least 100 cigarettes, or 50 cigars, or 3 packages of pipe tobacco over their lifetime. A positive history of diabetes was defined by an affirmative answer to the question “Has a doctor or other health professional ever said you have diabetes, glucose intolerance, or high blood sugar?” A positive history of hypertension was defined as an affirmative answer to the question “Has a doctor or other health professional ever said you had high blood pressure or hypertension?”

### Postural sway testing

Center of mass (COM) sway was measured in the anterior-posterior and mediolateral directions using BalanSens™ (BioSensics LLC, Brookline, MA) which is highly correlated with center of pressure sway area (Najafi et al., 2010). Participants stood with feet together and arms at their sides, first on the floor first with eyes open (FLEO) and then with eyes closed (FLEC) and then they stood on a foam cushion (Sunmate, Dynamic Systems, Inc.) of density 72.2 kg/m^3^ first with eyes open (FOEO) and then with eyes closed (FOEC). Participants were provided up to three attempts to successfully complete one trial lasting 40 seconds for each Condition (Wu et al., 2009; De Nunzio et al., 2014). Participants were excluded from postural sway testing if they required assistance to stand from sitting or to walk to minimize the risk of falling. Participants were progressed from Condition 1 through 4, and in this cohort all participants were able to complete the 1st three Conditions and progress to Condition 4. Not all participants were able to successfully complete Condition 4. Sway data was corrupt or missing for 3 participants from the floor eyes open test. The 95% confidence interval for COM sway area was calculated using custom functions in MATLAB (MathWorks, Inc.) from the anterior-posterior and mediolateral COM displacement trajectories (Duarte et al., 2011).

### Visual testing

Visual fields were measured using a Humphrey single intensity (24 dB) full field (60°) screen (Humphrey Field Analyzer, Carl Zeiss Meditec, Dublin, CA). Monocular visual fields were measured, and from these data binocular visual fields were estimated from the composite of the more sensitive of the visual field locations from each eye (Nelson-Quigg et al., 2000). The composite binocular visual field was scored as number of points missed (out of a possible 96 points) on the visual field exam. The visual fields were separated into three areas: the central (56 points), upper peripheral (18 points), and lower peripheral fields (22 points). The central field corresponds to ~20° of visual field. The percentage of points missed in the lower peripheral field (VISFIELD) was used in all analyses as impaired lower visual fields have specifically been associated with increased postural sway (Black et al., 2008).

### Vestibular function tests

The vestibular system consists of three semicircular canals (horizontal, anterior and posterior) which detect rotations of the head, and two otolith organs (the saccule and utricle) which sense linear movements of the head and the orientation of the head with respect to gravity. Individuals participating in the BLSA who consent to participate in vestibular testing underwent tests for both semicircular canal function (head impulse test) and otolith function [cervical and ocular vestibular evoked myogenic potentials (VEMPS)] as described below.

#### Video head impulse testing

Methods to measure horizontal semicircular canal function have been published previously and validated in older adults (Bartl et al., 2009; MacDougall et al., 2009; Agrawal et al., 2014). In brief, participants wore the EyeSeeCam video-oculography system, a lightweight goggle frame with a built in camera to record right eye movements and an accelerometer to record head movement at a sampling frequency of 220 Hz (Interacoustics USA, Eden Prairie, MN). Participants sat ~1.25 m from a visual fixation target on the wall. Trained examiners tilted the participant's head 30° below horizontal to bring the horizontal semicircular canal into the plane of head rotation and then performed 10–15 small amplitude (15–20°) head impulses to the right and left, with peak velocity typically from 150° to 250° per second.

Horizontal vestibulo-ocular reflex (VOR) gain was calculated as the ratio of the eye velocity and head velocity 60 ms after the onset of the head impulse (Agrawal et al., 2014). We used a VOR gain < 0.8 to define vestibular hypofunction. Semicircular canal function (CANAL) was categorized using the following scale: normal = 0 (both ears VOR gain ≥ 0.8), unilateral semicircular canal loss = 1 (one ear VOR gain < 0.8) and bilateral semicircular canal loss (both ears VOR gain < 0.8). Participants were excluded from head impulse testing if they had restricted neck rotation or pain with neck rotation.

#### Vestibular evoked myogenic potential (VEMP) recording conditions

VEMP tests were performed to measure otolith function. The cervical VEMP (cVEMP) is considered a test of saccular function, while the ocular VEMP (oVEMP) is considered a measure of utricular function. Both tests together measure the combined function of the otolith system. A commercial electromyographic (EMG) system (Carefusion Synergy, software version 14.1, Dublin, OH, USA) was used to record EMG signals with disposable, self-adhesive, pregelled, Ag/AgCl electrodes with 40-inch safety leadwires from GN Otometrics (Schaumburg, IL, USA). EMG signals were amplified 2500x and band-pass filtered, 20–2,000 Hz for cervical VEMPs (Nguyen et al., 2010).

##### Ocular VEMPs

Subjects reclined with their upper bodies elevated at 30° from horizontal. The skin overlying both cheeks and the manubrium sterni was cleansed with alcohol preps before electrode placement. A non-inverting electrode was placed on the cheek ~3 mm below the eye, directly beneath the pupil. An inverting electrode was placed 2 cm below the non-inverting electrode, and a ground electrode was placed on the manubrium sterni. Before stimulation, participants were instructed to perform 20° vertical saccades to ensure that symmetrical signals were recorded from both eyes. Participants were instructed to maintain a 20° upward gaze during ocular VEMP (oVEMP) stimulation and recording. Midline vibration stimuli consisted of head taps delivered manually with an Aesculap model ACO12C reflex hammer fitted with an inertial microswitch trigger. Head taps were delivered at Fz, in the midline at the hairline, 30% of the distance between the inion and nasion. Fifty sweeps for head taps were averaged for each test. The oVEMP waveform consists of a negative peak (n10), identified as the first distinctive peak in the waveform, followed by a positive peak (p16), identified as the first distinctive trough in the waveform. Individuals with EMG recordings lacking definable n10 waves were defined as having an absent oVEMP response. oVEMP function was dichotomized as present (response in one or both ears) or bilaterally absent. Participants were excluded from the oVEMP test if they could not see the target.

##### Cervical VEMPs

Participants reclined with their upper bodies elevated at 30° from horizontal. A non-inverting electrode was placed at the midpoint of the sternocleidomastoid (SCM) muscle, an inverting electrode was placed on the sternoclavicular junction, and a ground electrode was placed on the manubrium sterni. Participants were instructed to lift their heads up from the head rest to provide tonic background SCM activity during stimulation and recording, and a pre-stimulus rectifying surface EMG signal of at least 50 μV over 10 ms was required for accepting a cervical VEMP (cVEMP) tracing. Air-conducted sound stimuli consisted of 500 Hz, 125 dB SPL tone bursts of positive polarity, with a linear envelope (1 ms rise/- fall time, 2 ms plateau), at a repetition rate of 5 Hz. Sound stimuli were delivered monaurally through Audiocups noise-excluding headset enclosures (Amplivox, Eden Prairie, MN). The cVEMP waveform consists of a positive peak (p13), identified as the first distinctive trough in the waveform, followed by a negative peak (n23), identified as the first distinctive peak in the waveform. Subjects with EMG recordings lacking definable p13 waves were defined as having an absent cVEMP response. cVEMP function was dichotomized as present (in one or both ears) or bilaterally absent. Participants were excluded from the cVEMP test if they had pain with turning their head fully to the side.

Overall otolith function (OTOLITH) was categorized using the following scale: 0 represented complete or partial saccular and utricular function bilaterally; 1 represented either saccular or utricular function was absent bilaterally; and 2 represented both saccular or utricular function were absent bilaterally.

### Proprioception testing

Proprioception threshold (PROP) at the ankle has been detailed previously and is described here in brief (Ko et al., 2015). Participants sat blindfolded on a chair with their right foot on a motorized pedal connected to a potentiometer measuring angular position of the ankle joint. The threshold test identified the minimal angular displacement (degrees) required for correct perception of passive movement direction (plantar flexion or dorsiflexion) at an angular speed of 0.3°/s. Participants pushed a button to indicate perception of ankle motion and verbally indicated the direction of rotation. The testing followed the pre-set sequence of ankle plantar flexion, dorsiflexion, dorsiflexion, and plantar flexion. The average of the angular displacement for the last two tests was used as the proprioception threshold (Ko et al., 2015). Higher values on threshold testing correspond to less sensitive ankle proprioception.

### Data analysis

First, a one way ANOVA compared the sway area across the four conditions. Second, simple linear regressions were used to separately examine the association between age, gender, VISFIELD, PROP, CANAL, and OTOLITH with the continuous dependent variable COM sway area. Initial models included BMI as a covariate, but since BMI was not significant in any multivariate model (data not shown). Additionally, initial multivariate models also included variables for hypertension, diabetes, and central nervous system disease. However, the presence of the disease state was not significantly associated with sway area (data not shown). Adding terms to account for BMI, hypertension, diabetes, and central nervous system disease to the regression model did not appreciably change the results; therefore they were not included in the final model. Next, a multivariate linear model regressing the continuous dependent variable COM sway area on PROP, CANAL, VISFIELD, and OTOLITH while controlling for age and gender was tested. VISFIELD was only included in models for balance conditions when the eyes were open. Loss of function for CANAL and OTOLITH variables were treated as categorical and compared to individuals with normal function as a reference. STATA 14 (College Station, TX, USA) was used for all analyses. Standardized betas were calculated for each multi-sensory regression using the command “beta” in STATA. The “beta” command normalizes all variables using the ratio of the standard deviation of the independent variables to the standard deviation of the dependent variable. Each multivariate analysis was considered independent; therefore, statistical significance was defined at α = 0.05.

## Results

**Figure 1:**
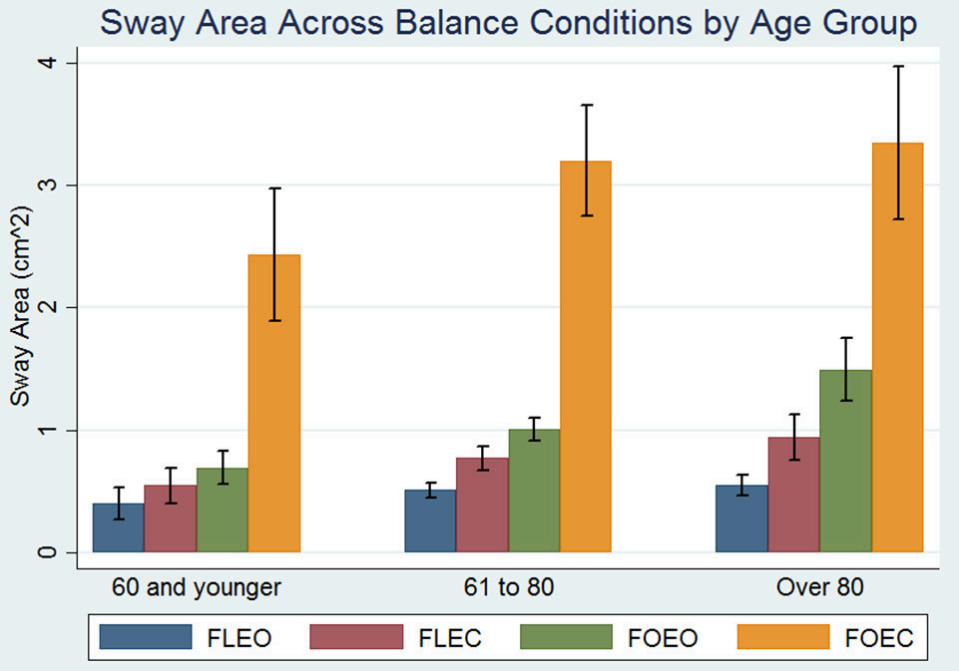
Effect of balance testing condition on the association between age and sway area. Blue bars represent mean sway area on the floor with eyes open (FLEO), purple bars represent mean sway area on the floor with eyes closed (FLEC), green bars represent mean sway area on foam with eyes open (FOEO), and orange bars represent mean sway area on foam with eyes closed (FLEC). Error bars represent the standard deviation. There were 43 participants under 60, 208 participants between 60 and 80, and 115 participants over the age of 80.

Participant demographics are presented in Table 1. All subjects were able to complete the FLEO, FLEC, and FOEO tests. Three hundred and twenty-two of Three hundred and sixty-six participants (87.9%) successfully completed the foam eyes closed test. Of the participants younger than 60 only 1 had absent single OTOLITH function, 2 had unilateral CANAL hypofunction, 3 missed 1 point on the visual fields exam, and none had a proprioception threshold that exceeded 1 *SD* of the sample average. The mean age of participants who successfully completed the FOEC test was 71.6 [(±12.5), range 27–93] and 55% of the participants (*n* = 177) were female. The mean age of participants who did not complete the FOEC test was 80.7 [(±9.6), range 32–93] and 51% of the participants (*n* = 24) were female. The mean sway area for each condition is presented by age groups [under 60 (*n* = 43), 60–80 (*n* = 208), and over 80 (*n* = 115)] in Figure 1. Sway area increased with age across all conditions in simple regressions. Sway area significantly increased as the sensory challenge increased, all pairwise comparisons *p*'s < 0.008.

**Table 1:** Participant demographics.

**Participant characteristics**	***N* or *Mean* (*SD*)**
Age	72.7 (12.6)
Gender	
Female	199
Male	167
History of smoking	160 (43.6%)
Hypertension	182 (50.1%)
Diabetes	77 (21.3%)
CNS disease	25 (6.9%)
VISFIELD (%)	2.23 (5.9), *n* = 321
PROP (°)	1.52 (1.7), *n* = 345
CANAL	
Normal: 0	*n* = 253
Unilateral loss: 1	*n* = 54
Bilateral loss: 2	*n* = 17
OTOLITH	
Normal: 0	*n* = 303
Any bilateral absent: 1	*n* = 51
Both bilateral absent: 2	*n* = 15
Sway area (cm^2^)	
FLEO	0.51 (0.43), *n* = 363
FLEC	0.80 (0.80), *n* = 366
FOEO	1.12 (0.99), *n* = 366
FOEC	3.14 (2.97), *n* = 322

*Average age and sensory function for the cohort*.

### Bivariate sway relationships

In the FLEO condition, VISFIELD was significantly associated with COM sway area (β = 0.01, *p* = 0.005); see Table 2 for following associations. PROP was also significantly associated with COM sway area while standing on FLEO (β = 0.08, *p* < 0.001). Age was significantly associated with increased sway (β = 0.005, *p* = 0.003).

**Table 2:** Bivariate relationships between COM sway area and sensory function, age, and gender.

**Balance conditions**	**FLEO**		**FLEC**		**FOEO**		**FOEC**	
**Predictor variables**	**β**	**95% CI**	**β**	**95% CI**	**β**	**95% CI**	**β**	**95% CI**
Age	0.005^*^	[0.002, 0.009]	0.01^*^	[0.006, 0.02]	0.02^*^	[0.02, 0.03]	0.033^*^	[0.01, 0.06]
Gender								
Female	Ref	Ref	Ref	Ref	Ref	Ref	Ref	Ref
Male	0.07	[−0.02, 0.16]	0.24^*^	[0.07, 0.40]	0.17	[−0.04, 0.37]	0.70^*^	[0.05, 1.35]
VISFIELD	0.01^*^	[0.003, 0.02]	N/A	N/A	0.02^*^	[0.003, 0.04]	N/A	N/A
PROP	0.08^*^	[0.05, 0.10]	0.13^*^	[0.08, 0.18]	0.20^*^	[0.14, 0.26]	0.052	[−0.16, 0.26]
CANAL								
Normal: 0	Ref	Ref	Ref	Ref	Ref	Ref	Ref	Ref
Unilateral loss: 1	0.06	[−0.10, 0.22]	0.33^*^	[0.03, 0.63]	0.36^*^	[0.01, 0.70]	0.38	[−0.74, 1.50]
Bilateral loss: 2	0.16	[−0.06, 0.39]	0.16	[−0.25, 0.58]	0.004	[−0.47, 0.48]	2.27^*^	[0.52, 4.02]
OTOLITH								
Normal: 0	Ref	Ref	Ref	Ref	Ref	Ref	Ref	Ref
Single otolith loss: 1	−0.03	[−0.16, 0.10]	−0.11	[−0.35, 0.14]	0.14	[−0.15, 0.44]	0.38	[−0.56, 1.31]
Both otolith loss: 2	−0.10	[−0.33, 0.12]	−0.40	[−0.81, 0.02]	0.11	[−0.42, 0.64]	0.32	[−1.24, 1.87]

Significant results indicated by

* *p < 0.05. FLEO, floor eyes open; FLEC, floor eyes closed; FOEO, foam eyes open; FOEC, foam eyes closed; PROP, proprioception threshold (degrees); Ref, Reference; VISFIELD, % loss lower visual field*.

In the FLEC condition, PROP was significantly associated with COM sway area (β = 0.13, *p* < 0.001). Individuals with unilateral CANAL loss had significantly increased COM sway area relative to individuals with normal CANAL function (β = 0.33, *p* = 0.031). Age was significantly associated with increased sway (β = 0.012, *p* < 0.001). Males swayed more than females (β = 0.24, *p* = 0.005).

In the FOEO condition, VISFIELD was significantly associated with COM sway area (β = 0.02, *p* = 0.021). PROP was significantly associated with COM sway area (β = 0.2, *p* < 0.001). Individuals with unilateral CANAL loss had significantly higher COM sway area relative to individuals with normal CANAL function (β = 0.36, *p* = 0.042). Age was significantly associated with increased sway (β = 0.024, *p* < 0.001).

In the FOEC condition, individuals with bilateral CANAL loss had significantly increased COM sway area relative to individuals with normal CANAL function (β = 2.3, *p* = 0.011). Age was significantly associated with increased sway (β = 0.033, *p* = 0.012). Males swayed more than females (β = 0.70, *p* = 0.035).

### Multi-sensory sway relationships

In the multi-sensory linear regression model for the condition FLEO, PROP (β = 0.09, *p* < 0.001) was significantly associated with COM sway area, see Table 3. Individuals with any complete OTOLITH function loss had significantly lower COM sway area relative to individuals with preserved OTOLITH function (β = −0.18, *p* = 0.035).

**Table 3:** Relationship between COM sway area and multi-sensory function controlling for age and gender.

**Balance test**	**FLEO (*****n*** = **246)**		**FLEC (*****n*** = **279)**		**FOEO (*****n*** = **248)**		**FOEC (*****n*** = **244)**	
**Predictor variables**	**β**	**95% CI**	**β**	**95% CI**	**β**	**95% CI**	**β**	**95% CI**
Age	0.002	[−0.002, 0.007]	0.009^*^	[0.001, 0.02]	0.02^*^	[0.006, 0.03]	0.025	[−0.004, 0.05]
Gender								
Female	Ref	Ref	Ref	Ref	Ref	Ref	Ref	Ref
Male	0.09	[−0.01, 0.2]	0.25^*^	[0.05, 0.45]	0.16	[−0.08, 0.39]	0.57	[−1.02, 3.01]
VISFIELD	0.005	[−0.01, 0.02]	N/A	N/A	0.005	[−0.02, 0.03]	N/A	N/A
PROP	0.09^*^	[0.05, 0.13]	0.13^*^	[0.07, 0.19]	0.19^*^	[0.11, 0.27]	−0.06	[−0.32, 0.20]
CANAL								
Normal	Ref	Ref	Ref	Ref	Ref	Ref	Ref	Ref
Unilateral reduction	0.04	[−0.13, 0.21]	0.22	[−0.08, 0.52]	0.32	[−0.05, 0.68]	0.18	[−0.92, 1.27]
Bilateral reduction	−0.03	[−0.27, 0.21]	−0.12	[−0.54, 0.30]	−0.36	[−0.88, 0.15]	2.12^*^	[0.40, 3.83]
OTOLITH								
Normal	Ref	Ref	Ref	Ref	Ref	Ref	Ref	Ref
Single otolith loss	−0.18^*^	[−0.34, −0.01]	−0.18	[−0.47, 0.11]	−0.16	[−0.51, 0.20]	0.31	[−0.70, 1.33]
Both otolith loss	−0.24	[−0.54, 0.05]	−0.48	[−1.01, 0.05]	−0.22	[−0.88, 0.44]	0.46	[−1.32, 2.23]

Not all participants completed all sensory tests, the sample size for each balance condition is indicated in the table. Significant results indicated by

* *p < 0.05. FLEO, floor eyes open; FLEC, floor eyes closed; FOEO, foam eyes open; FOEC, foam eyes closed; PROP, proprioception threshold (degrees); Ref, Reference; VISFIELD, % loss lower visual field*.

In the multi-sensory linear regression model for the condition FLEC, PROP (β = 0.13, *p* < 0.001) was significantly associated with COM sway area. Age (β = 0.009, *p* = 0.025) and gender (β = 0.25, *p* = 0.015) were also significantly associated with COM sway area while standing on FLEC.

In the multi-sensory linear regression model for the condition FOEO, PROP (β = 0.19, *p* < 0.001) was significantly associated with COM sway area. Age (β = 0.02, *p* = 0.0001) was also significantly associated with COM sway area while standing on FOEO.

**Figure 2:**
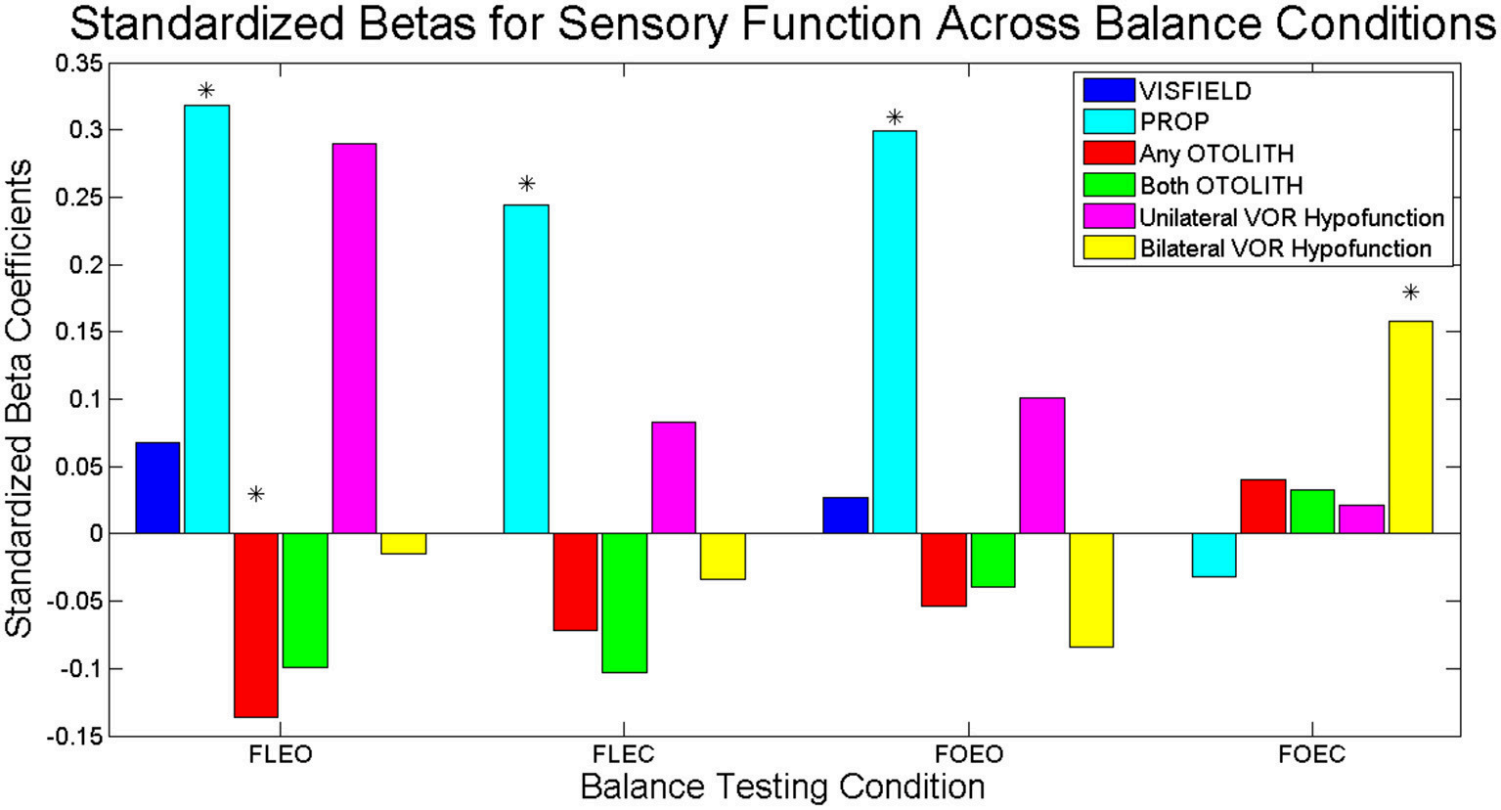
Standardized Betas for sensory contributions to sway area for each balance task. Dark blue bars represent the relative association of VISFIELD to sway area when vision was available (FLEO, FOEO). Light blue bars represent the relative association of PROP to sway area. Red bars represent the relative association of *any bilateral* OTOLITH loss to sway area. Green bars represent the relative association of *both bilateral* OTOLITH loss to sway area. Magenta bars represent the relative association of *unilateral* CANAL hypofunction to sway area. Yellow bars represent the relative association of *bilateral* CANAL hypofunction to sway area. Significant predictors from the multi-sensory models indicated with ^*^.

In the multi-sensory linear regression model for the condition FOEC, only CANAL function was significantly associated with COM sway area. Specifically bilateral CANAL loss was significantly associated with a greater COM sway area compared to individuals with normal canal function (β = 2.12, *p* = 0.016). To show the relative importance of sensory predictors within each balance condition, standardized β's are presented in Figure 2. There is a clear shift in the sensory system which contributes most to sway area when comparing Conditions 1–3 with Condition 4. PROP was the largest predictor of sway area until Condition 4 when bilaterally absent CANAL function became the largest predictor of sway area.

## Discussion

Our study builds on previous studies demonstrating that peripheral sensory function is the most important determinant of static postural stability in healthy older adults (Lord et al., 1991; Deshpande et al., 2016). We observed that ankle proprioceptive sensitivity was the dominant sensory predictor of increased postural sway in Conditions 1–3. However, bilaterally reduced semicircular canal function was the only significant predictor of COM sway in Condition 4. Both proprioceptive and vestibular function are known to decline as part of the healthy aging process (Ko et al., 2015; Li et al., 2015a,b). Increased weighting of proprioceptive input has also been reported for older adults relative to young adults regardless of health status (Pasma et al., 2015). These results suggest that independent of age, decline in proprioceptive function is a predominant contributor to the increased postural sway in older adults in most standing conditions.

We extend prior work by demonstrating that reduced vestibular function becomes the primary driver of increased postural sway in standing conditions with limited sensory information. We used rigorous vestibular physiologic tests in this study developed in the last few years, as opposed to more general balance measures that were previously used as a proxy for vestibular function (Lord et al., 1991). Notably, we were able to specifically observe a significant relationship between postural sway and semicircular canal function but not otolith function in Condition 4. The semicircular canals and otolith organs serve distinct functions: the canals are involved in detecting angular movements of the head while the otoliths sense the orientation of the head with respect to gravitational vertical. During standing the angular velocity of the head at the top of the inverted pendulum may be a more meaningful input signal than the corresponding deviation from the gravitational vector. Interestingly, any bilateral loss of OTOLITH function (either saccule or utricle) resulted in *less* sway, and only in the FLEO condition. However, the magnitude of this effect was less than half of the effect PROP had on sway area (see the magnitude of the standardized β coefficients in Figure 2). Although only a minor contributor in these analyses, OTOLITH function may be related to other aspects of postural control not captured by sway area. Future studies should investigate the effect of isolated loss of OTOLITH on multisensory reweighting during standing.

Consistent with previous reports, we observed that increased age was associated with increased sway (Choy et al., 2003; Illing et al., 2010). However, in two of the four multisensory models evaluated here, age was no longer a significant contributor to postural sway after accounting for sensory function. This is consistent with a previous report from Lord et al. (1991). Instead, sensory function was more strongly related to postural sway suggesting that the relationship between sway area and age may reflect a decline in sensory function. The difference in how age was related to sway area across the conditions suggests that in certain contexts age directly impacts sensory integration. In prior studies, older adults coupled more strongly to visual scene motion than younger adults and the process of dynamic re-weighting took longer relative to young adults (Jeka et al., 2010). When vision is not available (e.g., in FLEC), or conflicts with proprioception (e.g., in FOEO), sensory reweighting may take longer to process for older adults resulting in increased sway (Wiesmeier et al., 2015).

Despite being highly associated with sway area in bivariate analyses, reduced lower visual field sensitivity was not a significant contributor in the multivariate models. It is possible that visual field function may not be the most important visual function variable contributing to postural control. Contrast sensitivity and visual acuity are additional tests of static visual capability which decline with age and have been associated with increased sway (Black et al., 2008; Illing et al., 2010). The visual field test, as administered in this study, can be thought of more as a test of visual position detection and as such may underestimate the actual relationship between visual sensory function and postural control. Postural sway has been demonstrated to be highly dependent on visual velocity rather than position (Jeka et al., 2004). Visual motion perception degrades with age, and this decline in sensitivity to visual motion cannot be explained solely by static tests such as contrast sensitivity (Snowden and Kavanagh, 2006). The timing of reweighting to altered visual motion dynamics is delayed in older adults (Jeka et al., 2010). Future studies should investigate whether perceptual tests which detect the minimum visual velocity threshold better predict postural sway compared to static tests of visual function like visual fields, contrast sensitivity, and visual acuity.

In bivariate relationships males swayed significantly more than females when the eyes were closed. This effect remained significant for the multi-sensory model, but only for the floor eyes closed condition. Prior work demonstrated that males swayed more than females (Farenc et al., 2003), and attributed this to differences in body morphology and muscle physiology. In contrast to our current results, others reported that the gender difference in sway was only for conditions when the eyes were open (Cruz-Gómez et al., 2011). While body morphology and muscle physiology may contribute to this difference, future studies should investigate the interaction vision and gender for postural sway.

The current results suggest that the ability to reweight sensory input for postural control was intact for the adults who successfully completed all 4 balance conditions. Each successive condition results in an increased balance challenge due to progressively reduced accurate sensory input. A comparison of the standardized β coefficients across the balance tasks suggests effective sensory reweighting across conditions whereby the relative weighting of sensory inputs shifts away from proprioception toward vestibular function as proprioceptive inputs become unreliable and visual inputs become unavailable. However, we note that direct measurement of dynamic sensory reweighting requires an experimental design involving dynamically changing sensory perturbations and measuring real-time dynamics of sensory contributions to balance (Jeka et al., 2010; Engelhart et al., 2014). Further studies employing these methods will be needed to confirm that the mechanisms of multi-sensory reweighting may indeed be preserved in healthy older adults.

## Limitations

These data are cross-sectional and cannot be used to support causal inferences between COM sway area and age, vestibular, or proprioceptive function. Further, we cannot determine from these data whether the larger COM sway area with increased sensory challenge in these healthy adults represents maladaptive postural control since no individuals included in this analysis fell. A separate analysis is being conducted to evaluate individuals who experienced loss of balance. Moreover, there are other contributors to postural stability that were not specifically captured in this study. Age-related delays in sensory signal transmission, sensory integration and processing, and/or transmission of motor commands are known contributors to postural control in older adults (Lord et al., 1991; Wiesmeier et al., 2015). Adequate strength to maintain balance against gravity and correct for balance deviations also plays an important role (Manchester et al., 1989; Lord et al., 1991). By adjusting for age in our analyses we attempted to account for some of these factors. An additional limitation of our study is that the sensory function tests employed here were performed under passive conditions and while the participant was seated or reclined. An alternate approach would be to perform sensory function tests during balance tasks (Naranjo et al., 2015, 2016), which may provide a better measure of how the sensory systems perform *online*. Finally, this sample is made up of relatively healthy individuals since they were all able to stand and walk unassisted and the results may not be generalizable to populations with greater sensory impairments such as individuals with peripheral neuropathy or vestibular disease. This may contribute to the finding (data not shown) that disease state did not significantly add to the regression models to explain COM sway.

## Conclusion

In this cohort of healthy adults, when proprioception was unreliable and vision was unavailable, rotational vestibular function dominated the sensory contributions to postural sway. Under all other sensory conditions, proprioceptive function appears to be the most critical to postural control, independent of age. COM sway area consistently increases with age across balance conditions; however, this effect appears to be mediated by sensory function.

## Author contributions

EA and YA conceived the study. EA and RB collected data. EA, BS, ND, and YA analyzed data. EA and YA drafted the manuscript. EA, RB, BS, ND, SS, JJ, YA edited the manuscript. YA provided funding support for the study.

### Conflict of interest statement

The authors declare that the research was conducted in the absence of any commercial or financial relationships that could be construed as a potential conflict of interest.
